# Nano-Pore Size of Alumina Affects Osteoblastic Response

**DOI:** 10.3390/ijms19020528

**Published:** 2018-02-09

**Authors:** Federico Mussano, Tullio Genova, Francesca Giulia Serra, Massimo Carossa, Luca Munaron, Stefano Carossa

**Affiliations:** 1 CIR Dental School, Department of Surgical Sciences, University of Turin, via Nizza 230, 10126 Turin, Italy; tullio.genova@unito.it (T.G.); carossamassimo@gmail.com (M.C.) stefano.carossa@unito.it (S.C.); 2 Department of Life Sciences and Systems Biology, UNITO, via Accademia Albertina 13, 10123 Turin, Italy; luca.munaron@unito.it; 3 Department of Mechanical and Aerospatial Engineering (DIMEAS), Politecnico di Torino, Corso Duca degli Abruzzi 24, 10129 Turin, Italy; francesca.g.serra@gmail.com; 4 Centre for Nanostructured Interfaces and Surfaces (NIS), via Quarello 11/A, 10135 Turin, Italy

**Keywords:** MC3T3 cells, nano-porous alumina, nanotexture, cell adhesion, cell viability, in vitro osteogenesis

## Abstract

The rapid development and application of nanotechnology to biological interfaces has impacted the bone implant field, allowing researchers to finely modulate the interface between biomaterials and recipient tissues. In the present study, oxidative anodization was exploited to generate two alumina surfaces with different pore diameters. The former displayed surface pores in the mean range of 16–30 nm, while in the latter pores varied from to 65 to 89 nm. The samples were characterized by Field Emission Scanning Electron Microscopy (FESEM) and Energy Dispersive X-ray spectroscopy (EDX) analysis prior to being tested with pre-osteoblastic MC3T3-E1 cells. In vitro cell response was studied in terms of early cell adhesion, viability, and morphology, including focal adhesion quantification. Both the alumina samples promoted higher cell adhesion and viability than the control condition represented by the standard culture dish plastic. Osteogenic differentiation was assessed through alkaline phosphatase activity and extracellular calcium deposition, and it was found that of the two nano-surfaces, one was more efficient than the other. By comparing for the first time two nano-porous alumina surfaces with different pore diameters, our data supported the role of nano-topography in inducing cell response. Modulating a simple aspect of surface texture may become an attractive route for guiding bone healing and regeneration around implantable metals.

## 1. Introduction

Modern oral dental implants have been developed since Brånemark’s first discovery and successful research line [1]. Titanium implants rely on high survival rates, allowing satisfactory and predictable clinical [2] outcomes. Despite its biocompatibility, adequate strength, and corrosion resistance, however, Ti may no longer be deemed a completely bioinert material: several studies pointed out its possible allergenic action [3,4,5]. In addition, remarkable titanium concentrations were dosed in the proximity of oral implants [5] and in regional lymph nodes [6], which might be hazardous to the human body. Recently, to address these issues, at least in part, massive ceramics such as yttria-stabilized zirconia [7,8] and alumina-toughened zirconia [9,10] composites have been introduced as alternative implant materials. Unfortunately, they do not seem to possess mechanical properties [11] comparable to those of titanium alloys. 

A growing number of elderly and fragile patients require implant treatment, but are affected by poor bone quality or impaired healing conditions. They represent a great medical need and urge further advancements in the field. Whatever the material [12] and the level of biocompatibility and mechanical strength displayed, the capacity of actively enhanced bone healing is still a matter of intense research. To date, and only in pre-clinical studies, bioactivity has mainly been achieved through the grafting/functionalization of organic bioactive molecules [13,14], which, however, limits greatly their clinical usage. On the other hand, surface modification that does not imply the release of growth factors or signaling molecules may sensibly ameliorate the performance of a given material [15,16,17,18,19,20,21]. 

The unprecedented ability to control and characterize materials on the nanometer scale has led to a rapid development of the so-called nanostructured surfaces, whose properties vary considerably from those of the corresponding untreated materials [22]. While micro-topographic features were described as cell response modulators in a rich body of literature [23] and accelerate osseointegration [24,25], the role of nano-topography on cell behavior has been acknowledged only recently [26,27,28]. To improve cell surface interaction, different materials have been successfully nanostructured, or, in other terms, have been endowed with surface components such as tubes, spheres, grains, and fibers in the range of 0.1 to 100 nm [29,30]. The huge progress of nano-fabrication techniques [31] has enabled unique opportunities to finely modulate the interface between biomaterials and recipient tissues. This is of the utmost relevance as cells sense their environment by forming focal adhesions in their lamellipodia and filopodia [32,33,34].

Nanomaterials can be prepared from many solid materials such as metals, ceramics, polymers, organic materials, and composites. Due to its high strength to weight ratio, aluminum metal (Al) has become a suitable candidate for several bio-engineering applications [35]. Its surface characteristics can also be easily modulated through electro-chemical oxidation, achieving a thick layer usually called anodic aluminum oxide (AAO). This porous structure, endowed with a high aspect ratio, is the result of a highly reproducible process of tuning diameters along with periodicity and density distribution of the nano-pores [36]. Porous alumina substrates have received growing attention as bone interfaces [37], since bone cells can adhere and spread throughout their interconnected pores. Several studies reported that nano-porous alumina sustained the attachment and differentiation of osteoblasts [38,39,40,41] and mesenchymal stem cells [42] in vitro. 

In this study, nano-porous alumina samples with different pore diameters were compared in terms of early cell adhesion, viability, and osteogenic potential. The authors aimed to ascertain a possible role of the nano texture alone in promoting different cell behaviors, in the absence of other variables. 

## 2. Results

### 2.1. Morphology and Elemental Composition

**Figure 1:**
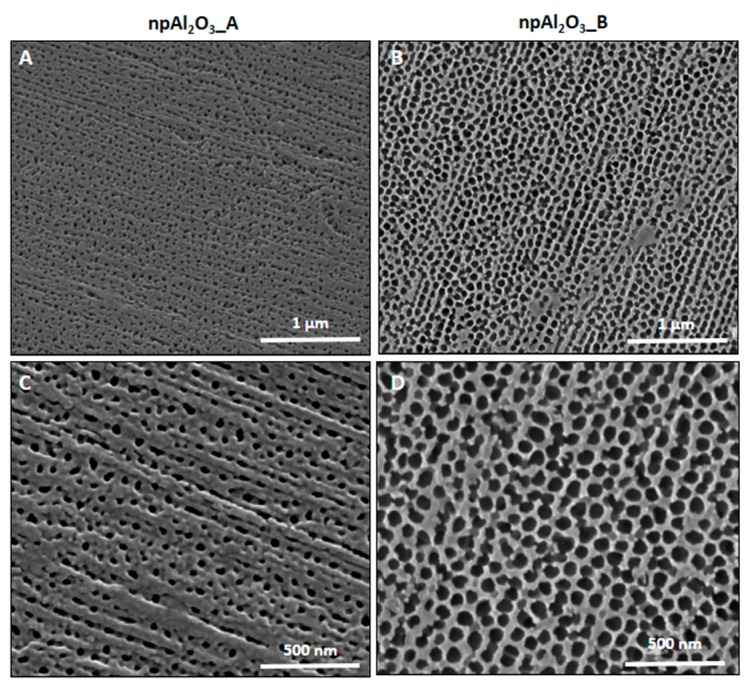
Electron microscopy. Field Emission Scanning Electron Microscope (FESEM) analysis of npAl_2_O_3__A (**A**,**C**) and npAl_2_O_3__B (**B**,**D**) at 100,000 (**A**,**B**) and 200,000 (**C**,**D**) magnifications.

**Figure 2:**
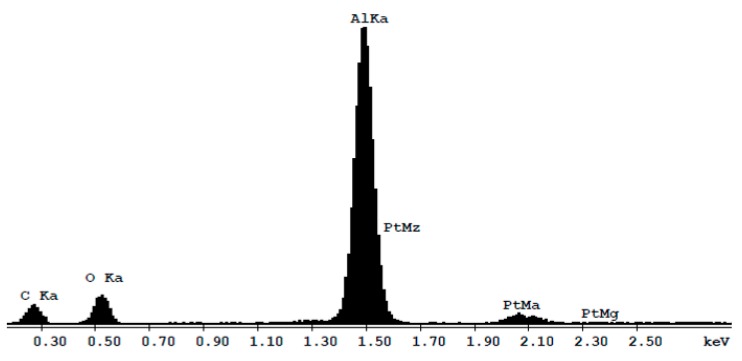
Energy Dispersive X-ray spectroscopy (EDX) of nano-porous Al_2_O_3_. Material is composed of aluminum (Al) and oxygen (O). The presence of platinum (Pt) is related to the metallization of the sample for FESEM analysis. The presence of the carbon (C) is due to sample contamination.

A view obtained by Field Emission Scanning Electron Microscopy (FESEM) of the nano-porous alumina surfaces, along with their cross-section, is given in Figure 1. As it can be observed, the samples differed in terms of pore size and thickness, which is also quantitatively reported in Table 1. As for the chemical composition of the specimens, it is portrayed in Figure 2 and Table 2.

### 2.2. Cell Adhesion

**Figure 3:**
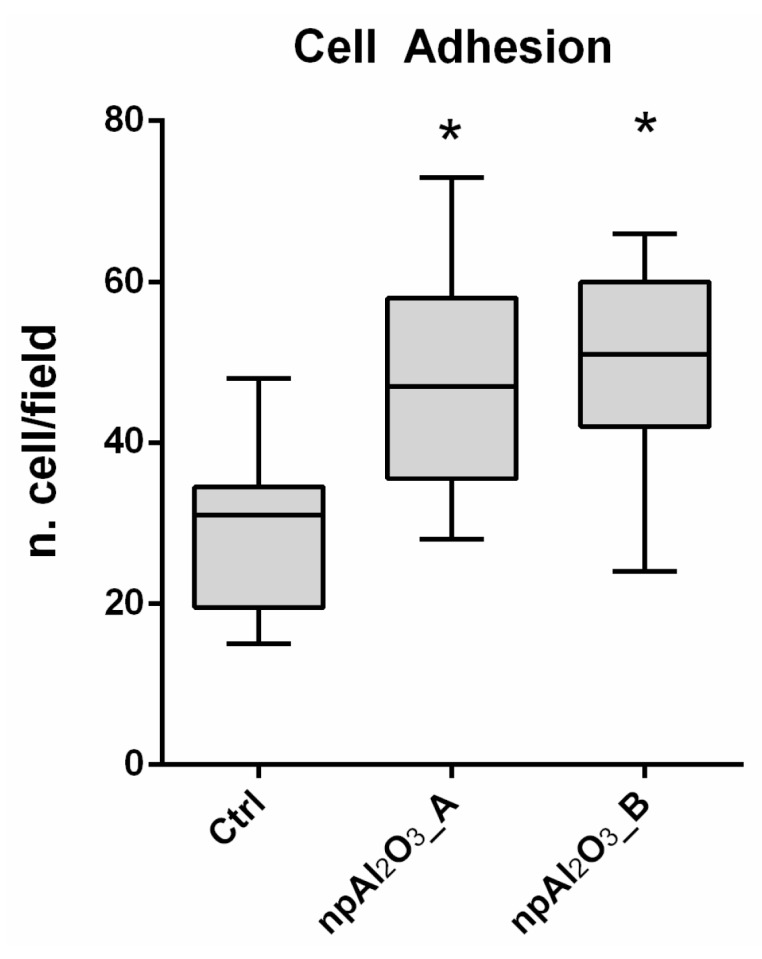
Cell adhesion evaluation. MC3T3-E1 adhesion was evaluated on all samples 10 min after seeding. The level of cell adhesion was measured by counting the number of nuclei for each field. The symbol (*) indicates a statistically significant difference versus the control (Ctrl), considering a *p*-value < 0.05.

In order to investigate the biological response elicited in vitro by the two different textures of nano-porous alumina, the widely diffused [43] pre-osteoblastic murine cell line MC3T3-E1 was used. Cells grown on the plastic dishes were used as a control. As it can be seen in Figure 3, both npAl_2_O_3__A and npAl_2_O_3__B significantly increased the number of adherent osteoblasts (10 min of seeding) compared to the control. 

### 2.3 Cell Viability

**Figure 4:**
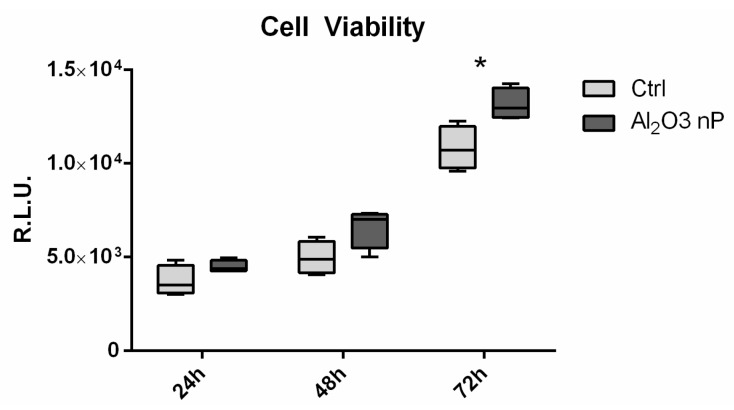
Cell viability evaluation. Cell viability of MC3T3-E1 performed through CellTiter-glo luminescent assay. Data are expressed as Relative Luminescent Unit (RLU) as measured at 24, 48, and 72 h after seeding. The symbol (*) indicates a statistically significant difference versus the control (Ctrl), considering a *p*-value < 0.05

Cell viability at 24, 48, and 72 h was similar in all of the tested conditions, albeit lower in the control at day 2 (Figure 4). At day 3, the cells plated on both of the np-alumina samples were significantly more viable than those on the control.

### 2.4. Cell Morphology and Focal Adhesion Quantification

**Figure 5:**
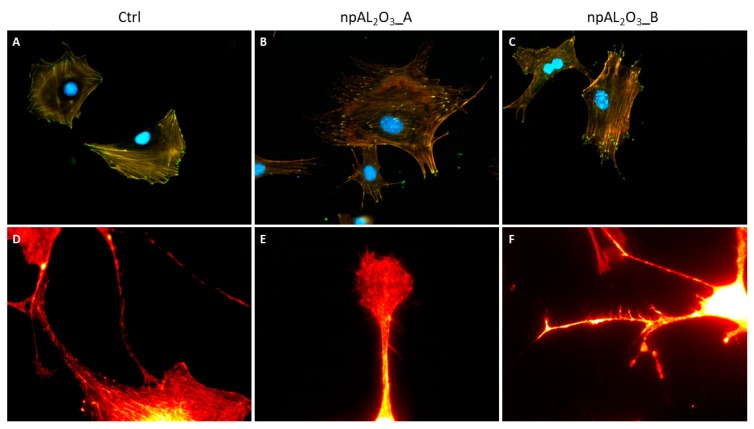
Representative pictures of MC3T3-E1 morphology. Fluorescence photomicrographs of MC3T3-E1 seeded on the control condition (**A**,**D**), npAl_2_O_3__A (**B**,**E**), and npAl_2_O_3__B (**C**,**F**). The cells were stained for the nucleus (DAPI, blue), the actin (rhodamine-phalloidin, red), and the focal adhesions (paxillin, green) at 200 magnifications (**A**–**C**) or only for actin at 900 magnifications (**D**–**F**).

MC3T3-E1 cells were properly adherent on control and nano-porous alumina samples at 24 h (Figure 5A–C). It is interesting to note that cells seeded on nano-porous alumina showed a very high number of filopodia and ramifications (Figure 5B,C,E,F) compared to cells seeded on the control material that appear more rounded and less elongated without ramifications (Figure 5A,D). 

**Figure 6:**
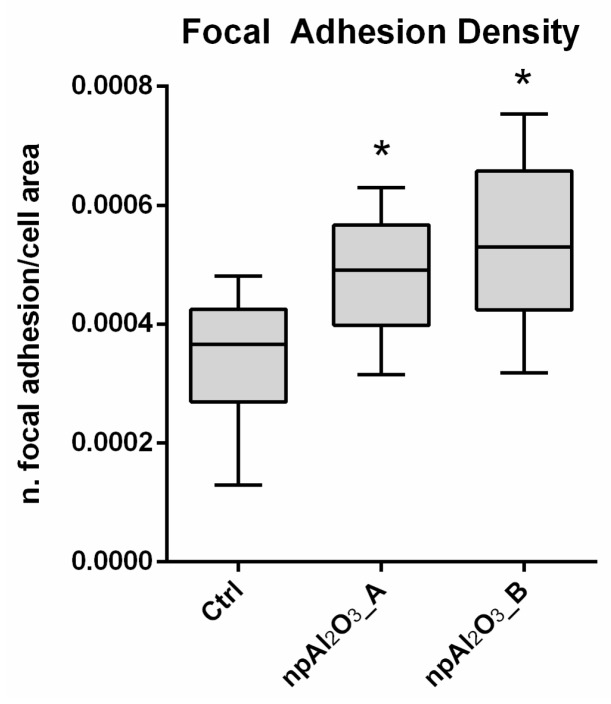
Focal adhesions density evaluation. Focal adhesions density was measured for MC3T3-E1 as the number of focal adhesions/cell area after 24 h from seeding on different samples. The symbol (*) indicates a statistically significant difference versus the control (Ctrl), considering a *p*-value < 0.05.

In addition, nano-porous alumina surfaces promoted significantly higher focal adhesion density than the control, as per the quantitative analysis (Figure 6).

### 2.5. Osteogenic Differentiation

**Figure 7:**
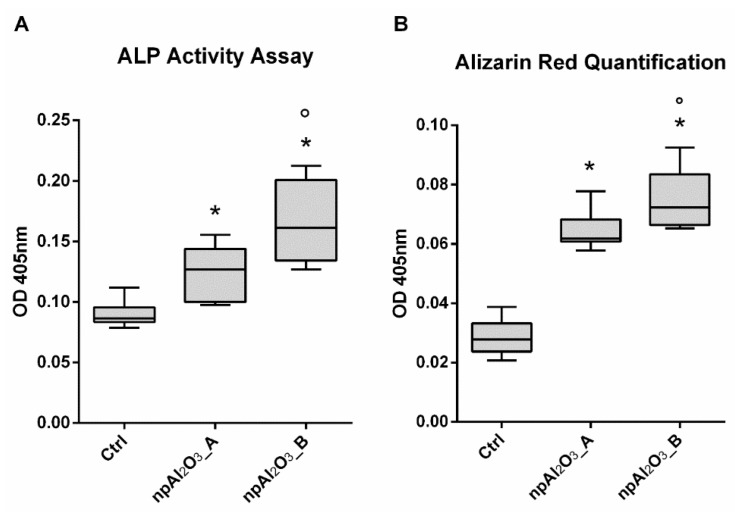
Osteogenic evaluation. Alkaline Phosphatase (ALP) activity assay (**A**) and Alizarin Red S quantification (**B**) performed by seeding MC3T3-E1 on different samples for 7 days (**A**) and for 21 days (**B**). The symbol (*) indicates a statistically significant difference versus the control (Ctrl), considering a *p*-value < 0.05. The symbol (°) indicates a statistically significant difference versus npAl_2_O_3__A, considering a *p*-value < 0.05.

The osteogenic differentiation was assessed by evaluating the Alkaline Phosphatase (ALP) activity at 7 days (Figure 7A) and the calcium deposition at 21 days (Figure 7B). Both nano-porous alumina surfaces significantly increased ALP activity and calcium deposition compared to the control condition; moreover, npAl_2_O_3__B showed significantly enhanced ALP activity and calcium deposition compared to npAl_2_O_3__A (Figure 7A,B). 

## 3. Discussion

Cell adhesion, proliferation, and differentiation result from the complex interaction between cells and the extracellular environment, where physicochemical cues trigger specific cellular processes owing to the spatially and temporally coordinated integration of outer stimuli performed by cells [22]. Among all the possible surface features, topography [24,25] at the dental implant interface has been recognized for many years as a factor of paramount relevance. The importance of nanoscale topography in guiding cell functions such as cell morphology, adhesion, and viability has been reported for endothelial cells grown on three different island heights of 13, 35, and 95 nm obtained by the demixing of polystyrene and poly (4-bromostyrene) in a paradigmatic study by Dalby and colleagues [44]. Titanium nano-surfaces [45] with a mean pore size of 20 nm were found to induce focal adhesions and filopodia in osteogenic cells.

To investigate the possible role played by the nano-topography of an acknowledged biocompatible material like alumina [46,47,48] on the early response and differentiation of pre-osteoblasts, we prepared two different nano-porous aluminum oxide surfaces that were chemically identical, but differed in pore size. Namely, npAl_2_O_3__A displayed surface pores in the mean range of 16–30 nm, while npAl_2_O_3__B pores varied from to 65 to 89 nm. Both samples supported cell adhesion and viability better than the control represented by the standard culture dish plastic, which is consistent with the literature [38,39]. 

Focal adhesions are the sites of interaction between the extracellular matrix and the cytoskeleton, mainly mediated through the integrin family [49]. To visualize these cell structures, the integrin-binding protein Paxillin was stained with immunofluorescence. Notably, the so-called focal adhesion density, i.e., the number of focal adhesions per surface unit, increased on both the alumina surfaces significantly compared to the control condition. Thus, the present work showed that the higher number of adherent and viable cells correlated with a significant increase in focal adhesion density, as observed elsewhere [45]. Nano-porous surfaces were capable of triggering the cellular mechanisms regulating the formation and maturation of the focal adhesions, which, in turn, could strengthen cell adhesion to the surface and possibly trigger intracellular cascades regulating cell behavior.

Interestingly, npAl_2_O_3__B promoted osteo-differentiation more effectively than npAl_2_O_3__A, as detected from alkaline phosphatase activity and calcium deposition in the mineralized matrix. In accordance, the nano-pores attained on titanium and Ti_6_Al_4_V—creating oxide surface layers [50]—selectively enhanced osteoblasts’ activity in vitro [51,52] and osteogenesis in a canine model [53]. Substrate nanofeatures have been also investigated as versatile tools for guiding either maintenance or differentiation in innovative culture systems [46,47,48], without falling back to the well-established usage of soluble chemical cues. 

It is to be underscored that only one study [54] directly compared the effects of pore diameters on late osteoblastic differentiation, and none have employed nano-porous alumina, to our knowledge. Specifically, Lavenus et al. [54] cultured human mesenchymal stem cells (hMSCs) on three nanostructured titanium substrates with 30, 150, and 300 nm pores, along with a suitable control. At 24 h, cells already exhibited different morphologies as a function of surfaces. Overall, the surface with the smallest nano-pores proved to be the most effective for the osteogenic differentiation of hMSCs. Our findings may postulate an optimal effect at a slightly different dimensional range, npAl_2_O_3__B being better than npAl_2_O_3__A, which seems surprisingly consistent with the data obtained by Nasrollahi and colleagues [55]. Indeed, these authors observed that anodized aluminum oxide membranes of 80 nm enhanced cell activity more than those with 40 nm pore diameters in NIH-3T3 fibroblast cells. 

The possible mechanisms underlying the correlation between pore size and enhanced cell response have been recently postulated [43], but they are far from having been completely dissected. Stimulating perspectives were offered by Song et al. [56] on the modulatory effects that macrophages grown on nano-porous anodic alumina exert on the osteogenic differentiation of bone marrow stromal cells (BMSCs). In their paradigmatic work [56], the authors proved that, among all the different pore sizes tested, the osteo-immune environment promoted by the 50 nm nano-porous structure was beneficial to the osteo-differentiation of BMSCs. Indeed, nano-topography and pore size affected macrophage spreading, shape, and activation, leading ultimately to the modulation of the inflammatory response and the release of osteogenic factors including bone morphogenetic protein 2 (bmp2) and WNT10b. Consequently, BMSCs treated with 50 nm nano-porous structure/macrophage-conditioned medium produced more mineralization nodules and expressed higher levels of collagen 1 and osteopontin than the conditioned medium obtained on polished substrates. Furthermore, media conditioned by the stimulated macrophages could upregulate the expression of three important osteogenic factors, namely, bmp2, bmp6, and the wingless-type MMTV integration site family. 

According to McMurray et al. modulating the substrate topography [47] and specifically its porosity could be the key to easily establishing culture systems aimed at maintaining a given cell population or to direct its lineage commitment, eventually producing new bioreactors. Alternatively, knowing the optimal nano-pore size for cell differentiation could be conveniently exploited whenever bone is needed in tissue engineering protocols. To follow this promising route, further research is required to elucidate properly how topography is capable of guiding osteo-differentiation both in vitro and in vivo on nanostructured interfaces.

## 4. Materials and Methods 

### 4.1. Sample Preparation

Nano-porous alumina was prepared and shaped into 10-mm diameter membranes by anodization in an acid environment. (Eltek SpA, Casale Monferrato, Italy).

### 4.2. Scanning Electron Microscopy and Energy Dispersive X-ray Spectroscopy

The microstructure of the samples was characterized through a Field Emission Scanning Electron Microscope (FESEM) (FEI INSPECT F, Thermo Fisher Scientific, Waltham, MA, USA) with an Energy Dispersive X-ray spectroscopy (EDX) analyzer for elemental composition analysis. Prior to being observed and tested, the samples were washed in distilled water, rinsed thoroughly in 70% ethanol, cleaned ultrasonically for 20 min in absolute ethanol, and finally air dried under a laminar flow hood to avoid contamination.

### 4.3. Biological Assays

To assess the biological response in vitro, the pre-osteoblastic murine cell line MC3T3-E1 (ECACC, Salisbury, UK) was used. Cells were maintained in Alpha Modified Eagle Medium (Alpha MEM) supplemented with 10% fetal bovine serum (Life Technologies, Milan, Italy), 100 U/mL penicillin, and 100 μg/mL streptomycin, under a humidified atmosphere of 5% CO_2_ in air, at 37 °C. To prevent contact inhibition, cells were passaged at subconfluency. 

#### 4.3.1. Cell Adhesion

Cell adhesion was evaluated on np-alumina samples using a 24-well plate (BD, Milan, Italy) as a support. After being detached with trypsin for 3 min, cells were counted and seeded at 1 × 103 cells/disk in 100 μL of growth medium on the np-alumina samples [57]. The 24-well plates were kept at 37 °C, 0.5% CO_2_ for 10 min. Before and after fixation in 4% paraformaldehyde in Phosphate Buffered Saline PBS for 15 min at room temperature, cells were washed two times with PBS and then stained with 1 μM DAPI (Molecular Probes, Eugene, CA, USA) for 15 min at 37 °C to visualize cell nuclei. Images were acquired using a Nikon Eclipse T-E microscope with a 40× objective. As previously reported [16,58,59,60], the cell nuclei were counted using the ‘Analyze particles’ tool of ImageJ software (ImageJ, U. S. National Institutes of Health, Bethesda, MD, USA, Available online: http://imagej.nih.gov/ij/).

#### 4.3.2. Cell Viability

MC3T3-E1 cells were plated at a density of 2500 cells/well in 24-well culture dishes and the proliferation rate was assessed by Cell Titer GLO (Promega, Milan, Italy) according to the manufacturer’s protocol at 1, 2, and 3 days. 

#### 4.3.3. Cell Morphology and Focal Adhesion Quantification

MC3T3-E1 cells were seeded at a concentration of 5000 cells/well in a 24-well plate. After 24 h, cells were fixed in 4% paraformaldehyde in Phosphate Buffer Saline (PBS) and stained with Rodamine-Phalloidin and DAPI (Life Technologies, Milan, Italy) to highlight the actin network and nuclei, respectively. Focal adhesions were specifically detected by an anti-Paxillin N-Term 04-581 antibody from Millipore (Merk, Darmstadt, Germany) [61]. Images were acquired with a Nikon Eclipse Ti-E microscope using different objectives: Nikon Plan 20×/0.10; Nikon Plan Fluor 40×/0.75; Nikon Plan Apo VC 60×/1.40 (Nikon Instruments, Amsterdam, The Netherlands). Cell spreading and focal adhesion density were quantified with ImageJ software.

#### 4.3.4. Osteogenic Cell Differentiation

To assess the osteogenic differentiation, MC3T3-E1 cells were cultured in osteogenic media by supplementing the normal culture medium with 10 mM β-glycerophosphate and 50 ng/mL ascorbic acid. 

#### 4.3.5. Alkaline Phosphatase Activity

Alkaline Phosphatase Activity (ALP) was determined colorimetrically as previously reported [62,63,64] and assessed at day 7. Cells were lysed with 0.05% Triton X-100 and incubated with the reagent solution containing phosphatase substrate (Sigma-Aldrich, Milan, Italy) at 37 °C for 15 min. A calibration curve of p-nitrophenol standards was always used. Alkaline phosphatase values were determined (Optical Density 405 nm) and normalized to the whole protein content, which was determined (Optical Density 562 nm) with a BCA™ Protein Assay (Thermo Fisher Scientific, Waltham, MA, USA). 

#### 4.3.6. Calcium Content 

The extracellular matrix calcification was quantified by Alizarin Red staining. At day 21, MC3T3-E1 cells were first incubated in a solution of 40 mM Alizarin Red (pH 4.2) and subsequently lysed with acetic acid. Absorbance of the lysates was finally measured at 405 nm.

### 4.4. Statistical Analysis

Data were analyzed by GraphPad Prism6 (GraphPad Software, Inc., La Jolla, CA, USA). Each experiment was repeated at least three times. Statistical analysis was performed by using the Mann-Whitney test. A *p*-value of < 0.05 was considered significant.

## Figures and Tables

**Table 1:** The pore size distribution over the cross-section of the sample and on the surface.

Surface Features		Sample A	Sample B
Pore distribution		Homogenous	Homogenous
Pore diameter on the surface		16–30 nm	65–89 nm
Pore diameter in the cross-section of the sample	Surface	16–30 nm	64–87 nm
	Center	16–20 nm	47–62 nm
	Substrate	10–20 nm	25–40 nm
Thickness		25 µm	82 µm

**Table 2:** Relative elemental concentrations found on the specimens are given in wt % (weight percent) and in at % (atomic percentage). Pt was used to metallize the samples before FESEM analysis.

Element	wt %	at %
C	39.39	55.78
O	20.38	21.26
Al	35.06	22.10
Pt	5.17	0.45
Total	100.00	100.00

## Abbreviations

Al	Aluminum metal
AAO	Anodic Aluminum Oxide
FESEM	Field Emission Scanning Electron Microscope
EDX	Energy Dispersive X-ray spectroscopy
PBS	Phosphate Buffer Saline
ALP	Alkaline Phosphatase Activity

## References

[1] Albrektsson T., Sennerby L. (1991). State of the art in oral implants. J. Clin. Periodontol..

[2] Lindquist L.W., Carlsson G.E., Jemt T. (1996). A prospective 15-year follow-up study of mandibular fixed prostheses supported by osseointegrated implants. Clinical results and marginal bone loss. Clin. Oral Implants Res..

[3] Evrard L., Waroquier D., Parent D. (2010). Allergies to dental metals. Titanium: A new allergen. Rev. Med. Brux..

[4] Pigatto P.D., Guzzi G., Brambilla L., Sforza C. (2009). Titanium allergy associated with dental implant failure: Letters to the Editor. Clin. Oral Implants Res..

[5] Sicilia A., Cuesta S., Coma G., Arregui I., Guisasola C., Ruiz E., Maestro A. (2008). Titanium allergy in dental implant patients: A clinical study on 1500 consecutive patients. Clin. Oral Implants Res..

[6] Onodera K., Ooya K., Kawamura H. (1993). Titanium lymph node pigmentation in the reconstruction plate system of a mandibular bone defect. Oral Surg. Oral Med. Oral Pathol..

[7] Depprich R., Naujoks C., Ommerborn M., Schwarz F., Kübler N.R., Handschel J. (2014). Current findings regarding zirconia implants. Clin. Implant Dent. Relat. Res..

[8] Andreiotelli M., Wenz H.J., Kohal R.J. (2009). Are ceramic implants a viable alternative to titanium implants? A systematic literature review. Clin. Oral Implants Res..

[9] Spies B.C., Sperlich M., Fleiner J., Stampf S., Kohal R.J. (2015). Alumina reinforced zirconia implants: 1-year results from a prospective cohort investigation. Clin. Oral Implants Res..

[10] Schierano G., Mussano F., Faga M.G., Menicucci G., Manzella C., Sabione C., Genova T., von Degerfeld M.M., Peirone B., Cassenti A. (2015). An Alumina Toughened Zirconia Composite for Dental Implant Application: In Vivo Animal Results. Biomed Res. Int..

[11] Cionca N., Hashim D., Mombelli A. (2017). Zirconia dental implants: Where are we now, and where are we heading?. Periodontology 2000.

[12] Duraccio D., Mussano F., Faga M.G. (2015). Biomaterials for dental implants: Current and future trends. J. Mater. Sci..

[13] Mandracci P., Mussano F., Rivolo P., Carossa S. (2016). Surface Treatments and Functional Coatings for Biocompatibility Improvement and Bacterial Adhesion Reduction in Dental Implantology. Coatings.

[14] Vallée A., Faga M.G., Mussano F., Catalano F., Tolosano E., Carossa S., Altruda F., Martra G. (2014). Alumina-zirconia composites functionalized with laminin-1 and laminin-5 for dentistry: Effect of protein adsorption on cellular response. Colloids Surf. B Biointerfaces.

[15] Mussano F., Genova T., Laurenti M., Munaron L., Pirri C.F., Rivolo P., Carossa S., Mandracci P. (2018). Hydrogenated amorphous silicon coatings may modulate gingival cell response. Appl. Surf. Sci..

[16] Mussano F., Genova T., Verga Falzacappa E., Scopece P., Munaron L., Rivolo P., Mandracci P., Benedetti A., Carossa S., Patelli A. (2017). In vitro characterization of two different atmospheric plasma jet chemical functionalizations of titanium surfaces. Appl. Surf. Sci..

[17] Mandracci P., Mussano F., Ceruti P., Pirri C.F., Carossa S. (2015). Reduction of bacterial adhesion on dental composite resins by silicon–oxygen thin film coatings. Biomed. Mater..

[18] Mussano F., Genova T., Rivolo P., Mandracci P., Munaron L., Faga M.G., Carossa S. (2017). Role of surface finishing on the in vitro biological properties of a silicon nitride–titanium nitride (Si3N4–TiN) composite. J. Mater. Sci..

[19] Gazia R., Mandracci P., Mussano F., Carossa S. (2011). AlNx and a-SiOx coatings with corrosion resistance properties for dental implants. Surf. Coat. Technol..

[20] Mandracci P., Ceruti P., Ricciardi C., Mussano F., Carossa S. (2010). a-SiO_X_ Coatings Grown on Dental Materials by PECVD: Compositional Analysis and Preliminary Investigation of Biocompatibility Improvements. Chem. Vap. Depos..

[21] Mandracci P., Mussano F., Ricciardi C., Ceruti P., Pirri F., Carossa S. (2008). Low temperature growth of thin film coatings for the surface modification of dental prostheses. Surf. Coat. Technol..

[22] Mendes P.M. (2013). Cellular nanotechnology: Making biological interfaces smarter. Chem. Soc. Rev..

[23] Wennerberg A., Albrektsson T. (2009). Effects of titanium surface topography on bone integration: A systematic review. Clin. Oral Implants Res..

[24] Morton D., Bornstein M.M., Wittneben J.-G., Martin W.C., Ruskin J.D., Hart C.N., Buser D. (2010). Early Loading after 21 Days of Healing of Nonsubmerged Titanium Implants with a Chemically Modified Sandblasted and Acid-Etched Surface: Two-Year Results of a Prospective Two-Center Study. Clin. Implant Dent. Relat. Res..

[25] Bornstein M.M., Schmid B., Belser U.C., Lussi A., Buser D. (2005). Early loading of non-submerged titanium implants with a sandblasted and acid-etched surface. Clin. Oral Implants Res..

[26] Dalby M.J., Gadegaard N., Oreffo R.O.C. (2014). Harnessing nanotopography and integrin–matrix interactions to influence stem cell fate. Nat. Mater..

[27] Rosa A.L., Kato R.B., Castro Raucci L.M.S., Teixeira L.N., de Oliveira F.S., Bellesini L.S., de Oliveira P.T., Hassan M.Q., Beloti M.M. (2014). Nanotopography Drives Stem Cell Fate Toward Osteoblast Differentiation Through α1β1 Integrin Signaling Pathway. J. Cell. Biochem..

[28] Puckett S., Pareta R., Webster T.J. (2008). Nano rough micron patterned titanium for directing osteoblast morphology and adhesion. Int. J. Nanomed..

[29] Srivatsan T.S. (2014). Biomaterials: A Nano Approach, by Sreeram Ramakrishna, Murugan Ramalingam, T.S. Sampath Kumar, and Winston O. Soboyejo. Mater. Manuf. Process..

[30] Greco R.S., Prinz F.B., Smith R.L. (2005). Nanoscale Technology in Biological Systems.

[31] Aguilar Z.P. (2012). Nanomaterials for Medical Applications.

[32] DeMali K.A., Wennerberg K., Burridge K. (2003). Integrin signaling to the actin cytoskeleton. Curr. Opin. Cell Biol..

[33] Hoffmann B., Schäfer C. (2010). Filopodial focal complexes direct adhesion and force generation towards filopodia outgrowth. Cell Adhes. Migr..

[34] Gallagher J.O., McGhee K.F., Wilkinson C.D.W., Riehle M.O. (2002). Interaction of animal cells with ordered nanotopography. IEEE Trans. Nanobiosci..

[35] Poinern G.E.J., Ali N., Fawcett D. (2011). Progress in Nano-Engineered Anodic Aluminum Oxide Membrane Development. Materials.

[36] Masuda H., Yada K., Osaka A. (1998). Self-Ordering of Cell Configuration of Anodic Porous Alumina with Large-Size Pores in Phosphoric Acid Solution. Jpn. J. Appl. Phys..

[37] Song Y., Ju Y., Morita Y., Xu B., Song G. (2014). Surface functionalization of nanoporous alumina with bone morphogenetic protein 2 for inducing osteogenic differentiation of mesenchymal stem cells. Mater. Sci. Eng. C.

[38] Karlsson M., Pålsgård E., Wilshaw P., Di Silvio L. (2003). Initial in vitro interaction of osteoblasts with nano-porous alumina. Biomaterials.

[39] Swan E.E.L., Popat K.C., Grimes C.A., Desai T.A. (2005). Fabrication and evaluation of nanoporous alumina membranes for osteoblast culture. J. Biomed. Mater. Res. Part A.

[40] Song Y., Ju Y., Morita Y., Song G. (2013). Effect of the nanostructure of porous alumina on growth behavior of MG63 osteoblast-like cells. J. Biosci. Bioeng..

[41] Ni S., Li C., Ni S., Chen T., Webster T.J. (2014). Understanding improved osteoblast behavior on select nanoporous anodic alumina. Int. J. Nanomed..

[42] Song Y., Ju Y., Song G., Morita Y. (2013). In vitro proliferation and osteogenic differentiation of mesenchymal stem cells on nanoporous alumina. Int. J. Nanomed..

[43] Genova T., Munaron L., Carossa S., Mussano F. (2016). Overcoming physical constraints in bone engineering: The importance of being vascularized. J. Biomater. Appl..

[44] Dalby M.J., Riehle M.O., Johnstone H., Affrossman S., Curtis A.S.G. (2002). In vitro reaction of endothelial cells to polymer demixed nanotopography. Biomaterials.

[45] Guadarrama Bello D., Fouillen A., Badia A., Nanci A. (2017). A nanoporous titanium surface promotes the maturation of focal adhesions and formation of filopodia with distinctive nanoscale protrusions by osteogenic cells. Acta Biomater..

[46] Park S.Y., Park J., Sim S.H., Sung M.G., Kim K.S., Hong B.H., Hong S. (2011). Enhanced Differentiation of Human Neural Stem Cells into Neurons on Graphene. Adv. Mater..

[47] McMurray R.J., Gadegaard N., Tsimbouri P.M., Burgess K.V., McNamara L.E., Tare R., Murawski K., Kingham E., Oreffo R.O.C., Dalby M.J. (2011). Nanoscale surfaces for the long-term maintenance of mesenchymal stem cell phenotype and multipotency. Nat. Mater..

[48] Park S.Y., Choi D.S., Jin H.J., Park J., Byun K.-E., Lee K.-B., Hong S. (2011). Polarization-Controlled Differentiation of Human Neural Stem Cells Using Synergistic Cues from the Patterns of Carbon Nanotube Monolayer Coating. ACS Nano.

[49] Gao C., Peng S., Feng P., Shuai C. (2017). Bone biomaterials and interactions with stem cells. Bone Res..

[50] Yi J.-H., Bernard C., Variola F., Zalzal S.F., Wuest J.D., Rosei F., Nanci A. (2006). Characterization of a bioactive nanotextured surface created by controlled chemical oxidation of titanium. Surf. Sci..

[51] De Oliveira P.T., Nanci A. (2004). Nanotexturing of titanium-based surfaces upregulates expression of bone sialoprotein and osteopontin by cultured osteogenic cells. Biomaterials.

[52] Nune K., Misra R., Gai X., Li S., Hao Y. (2017). Surface nanotopography-induced favorable modulation of bioactivity and osteoconductive potential of anodized 3D printed Ti-6Al-4V alloy mesh structure. J. Biomater. Appl..

[53] Tavares M.G., De Oliveira P.T., Nanci A., Hawthorne A.C., Rosa A.L., Xavier S.P. (2007). Treatment of a commercial, machined surface titanium implant with H_2_SO_4_/H_2_O_2_ enhances contact osteogenesis. Clin. Oral Implants Res..

[54] Lavenus S., Berreur M., Trichet V., Pilet P., Louarn G., Layrolle P. (2011). Adhesion and osteogenic differentiation of human mesenchymal stem cells on titanium nanopores. Eur. Cells Mater..

[55] Nasrollahi S., Banerjee S., Qayum B., Banerjee P., Pathak A. (2017). Nanoscale Matrix Topography Influences Microscale Cell Motility through Adhesions, Actin Organization, and Cell Shape. ACS Biomater. Sci. Eng..

[56] Chen Z., Ni S., Han S., Crawford R., Lu S., Wei F., Chang J., Wu C., Xiao Y. (2017). Nanoporous microstructures mediate osteogenesis by modulating the osteo-immune response of macrophages. Nanoscale.

[57] Canullo L., Genova T., Tallarico M., Gautier G., Mussano F., Botticelli D. (2016). Plasma of Argon Affects the Earliest Biological Response of Different Implant Surfaces. J. Dent. Res..

[58] Canullo L., Genova T., Mandracci P., Mussano F., Abundo R., Fiorellini J. (2017). Morphometric Changes Induced by Cold Argon Plasma Treatment on Osteoblasts Grown on Different Dental Implant Surfaces. Int. J. Periodontics Restor. Dent..

[59] Canullo L., Genova T., Wang H.-L., Carossa S., Mussano F. (2017). Plasma of Argon Increases Cell Attachment and Bacterial Decontamination on Different Implant Surfaces. Int. J. Oral Maxillofac. Implants.

[60] Genova T., Grolez G.P., Camillo C., Bernardini M., Bokhobza A., Richard E., Scianna M., Lemonnier L., Valdembri D., Munaron L. (2017). TRPM8 inhibits endothelial cell migration via a nonchannel function by trapping the small GTPase Rap1. J. Cell Biol..

[61] Avanzato D., Genova T., Fiorio Pla A., Bernardini M., Bianco S., Bussolati B., Mancardi D., Giraudo E., Maione F., Cassoni P. (2016). Activation of P2X7 and P2Y11 purinergic receptors inhibits migration and normalizes tumor-derived endothelial cells via cAMP signaling. Sci. Rep..

[62] Mussano F., Bartorelli Cusani A., Brossa A., Carossa S., Bussolati G., Bussolati B. (2014). Presence of osteoinductive factors in bovine colostrum. Biosci. Biotechnol. Biochem..

[63] Mussano F., Lee K.J., Zuk P., Tran L., Cacalano N.A., Jewett A., Carossa S., Nishimura I. (2010). Differential effect of ionizing radiation exposure on multipotent and differentiation-restricted bone marrow mesenchymal stem cells. J. Cell. Biochem..

[64] Mussano F., Genova T., Corsalini M., Schierano G., Pettini F., Di Venere D., Carossa S. (2017). Cytokine, Chemokine, and Growth Factor Profile Characterization of Undifferentiated and Osteoinduced Human Adipose-Derived Stem Cells. Stem Cells Int..

